# Comparison of two free light chain assays: performance of the involved free light chain ratio and implications for diagnosis of multiple myeloma

**DOI:** 10.1038/s41408-022-00722-5

**Published:** 2022-09-02

**Authors:** Maria Alice V. Willrich, David L. Murray, S. Vincent Rajkumar, Sandra C. Bryant, Dirk Larson, Vanessa Pazdernik, Melissa R. Snyder, Robert A. Kyle, Angela Dispenzieri

**Affiliations:** 1 Department of Laboratory Medicine and Pathology, Mayo Clinic, Rochester, MN US; 2 Division of Hematology, Mayo Clinic, Rochester, MN US; 3 Department of Quantitative Health Sciences, Division of Clinical Trials and Biostatistics, Mayo Clinic, Rochester, MN US

**Keywords:** Myeloma, Translational research

Dear Editor,

The free light chain (FLC) assay is a valuable tool to screen and prognosticate plasma cell disorders (PCDs), follow response to therapy and progression. The definition of multiple myeloma (MM) requiring therapy was updated in 2014 by the International Myeloma Working Group (IMWG) to include the use of the FLC as an independent criterion for diagnosis of MM even in the absence of hypercalcemia, renal insufficiency, anemia or bone lesions (CRAB), and patients with myeloma defining events (MDE: more than one bone lesion by MRI; bone marrow plasmacytosis of greater than 60%; or an involved to uninvolved FLC ratio (iFLCr) of 100 or more along with an involved FLC concentration (iFLC) of at least 100 mg/L were recategorized as MM instead of SMM [1]. The original studies that prompted the change in SMM and MM definitions found the iFLCr cutoff of 100 or more to be associated with a 97% specificity of progression to MM or AL at 2 years. All initial studies were done using the FreeLite^TM^ reagents (The Binding Site, Birmingham, UK). Other manufacturers have recently developed their own FLC assays. Sebia developed an FLC assay, which like the FreeLite assay uses polyclonal antibodies to FLCs. Unlike the FreeLite assay, which depends on nephelometry or turbidimetry, the Sebia assay is an ELISA method amenable to automation in multiple platforms without a change in how the samples are processed. It is known that there is a less than ideal linear correlation between the tests [2–4] (and our data is shown as supplemental materials), but little is known about the performance of the iFLCr cut-points between tests. In this work, we focus on two important FLC applications among patients with MM and smoldering multiple myeloma (SMM). The first relates to the diagnosis of MM; the second to the risk of SMM progression to MM using modern definitions.

From the original cohort of 586 newly diagnosed SMM patients seen at the Mayo Clinic from 1976 to 2011 [5, 6] with authorization to review their medical records for research purposes, we had access to 284 residual serum samples that met the 2003 IMWG criteria for SMM [7]. Aliquots of frozen stored serum were retrieved, and Sebia FLC tests were performed by ELISA. The ELISAs were run on a DS2 automated platform (Dynex Technologies, Chantilly, VA). FreeLite FLC results were from the medical record or research database from the 2008/2013 studies.

The median age of the cohort was 64 years, and 45.4% were female (Table 1). The median M-spike was 21.8 g/L (range 0 to 74.4 g/L) and the most common isotype of the monoclonal protein was IgG (72.2%). In the 284 patients, 137 (48.2%) progressed to MM and 5 (1.8%) to AL, with 85.4 (0.1, 462.9) median months of follow-up. Of those who did not progress, 67.6% (96/142) had died, precluding the possibility of further progression. We, therefore, tested the performance of both assays ability to predict progression at 2 years. The combination of iFLCr ≥100 and iFLC concentration ≥100 mg/L existed in 11.4% (30/263) patients when using the FreeLite assay and 8.8% (25/284) when using the Sebia assay (Table 2). The performance characteristics at 1 year, 2 years, and 5 years are shown in Table 2 and are quite comparable for the two tests (sensitivity and specificity at 2 years, both *p* ≥ 0.23), supporting that the iFLCr of 100 or more can be used for Sebia FLC with a sensitivity of 16.7% and specificity of 93.1%, virtually identical to FreeLite.

**Table 1:** Demographics of the SMM (2003 definition) cohort.

	Total (*N* = 284)
**Age**	
Median (Range)	64.0 (30.0–87.0)
**Sex**	
Female	129 (45.4%)
Male	155 (54.6%)
**Race**	
Caucasian	259 (91.2%)
Black	9 (3.2%)
Asian, American Indian & Other	4 (1.4%)
Unknown	12 (4.2%)
**Immunoglobulin heavy chain isotype**	
IgG	205 (72.2%)
IgA	61 (21.5%)
IgM	2 (0.7%)
IgD	1 (0.4%)
Biclonal	8 (2.8%)
Light chain only	7 (2.5%)
**Serum electrophoresis measured M-protein, g/L**	
Median (Range)	21.8 (0.0, 74.4)
M-protein >20 g/L, *n* (%)	160 (56.3%)
**Bone marrow plasma cells, %**^**a**^	
Median (Range)	25.0 (4.1–95.0)
≤60%	227 (95.8%)
>60%	10 (4.2%)
≤20%	102 (43.0%)
>20%	135 (57.0%)
**MRI identified bone lesions,** ***n*** **(%)**^**b**^	
Positive	10 (4.2%)
**eGFR, CKD-EPI equation, mL/min/1.73 m**^**2 c**^	
<30	12 (4.3%)
30–60	87 (31.1%)
>60	181 (64.6%)
**Free Light Chain Testing**^**d**^	

	Sebia		FreeLite		*P*
	*n* (%) or Median (Range)	*n*	*n* (%) or Median (Range)	*n*	
iFLCr > 20	101 (35.6%)	284	106 (40.3%)	263	0.10
iFLCr ≥ 100	26 (9.2%)	284	30 (11.4%)	263	0.30
iFLCr ≥ 100 and iFLC ≥ 100 mg/L	25 (8.8%)	284	30 (11.4%)	263	0.22
**For patients with a kappa involved light chain**^**e**^					
KFLC, mg/L	58.1 (0.0–3919)	190	59.5 (1.2–5370)	176	0.005
LFLC, mg/L	7.2 (0.0–2680)	190	8.6 (0.4–1450)	176	0.51
iFLCr	8.8 (0.0–1073)	190	9.8 (0.0–4526)	175	0.01
**For patients with a lambda involved light chain**^**e**^					
KFLC, mg/L	5.8 (1.6–43.1)	94	8.5 (0.3–112)	88	<0.0001
LFLC, mg/L	75.3 (14.6–7439)	94	117.8 (1.7–3880)	88	0.02
iFLCr	14.3 (1.0–592)	94	18.2 (1.0–11000)	88	0.26

IMWG 2003 definition of SMM (22): >10% BMPCs and/or serum M-protein ≥30 g/L, plus absence of CRAB attributable to a PCD (calcium >0.25 mmol/L above the reference interval or >2.75 mmol/L (>11.5 mg/dL), serum creatinine >173 µmol/L (>2 mg/dL), hemoglobin 2 g/dL below the reference interval or <10 g/dL, or lytic lesions or diffuse osteopenia).

^a^*N* = 237 because 47 patients did not have bone marrow biopsy performed within 30 days of SMM diagnosis date.

^b^*N* = 239 because 45 patients did not have an MRI measurement.

^c^*N* = 280 because four patients did not have a creatinine measurement.

^d^McNemar’s test for paired proportions. *N* = 263 because 21 patients did not have FreeLite involved FLC ratio.

^e^Paired Wilcoxon signed-rank test for KFLC, LFLC, and iFLCr. For kappa involved light chain: *N* = 176 because 14 did not have FreeLite KFLC; *N* = 176 because 14 did not have FreeLite LFLC; and *N* = 175 because 15 did not have FreeLite iFLCr. For lambda involved light chain: *N* = 88 because 6 did not have FreeLite KFLC; *N* = 88 because 6 did not have FreeLite LFLC; and *N* = 88 because 6 did not have FreeLite iFLCr.

**Table 2:** Performance (sensitivity and specificity analysis) of the involved FLC ratio cut-points for progression to active multiple myeloma or amyloidosis, and for high-risk smoldering multiple myeloma stratification, at interval time-points.

	*n* (%)	12 months, % (95% CI)	24 months, % (95% CI)	60 months, % (95% CI)
**iFLCr cut-points for progression to active multiple myeloma or amyloidosis,** ***n*** = **284**				
**Freelite cut-points**^**a**^				
*N* (%) of subjects who progressed		35 (13.3%)	65 (24.7%)	107 (40.7%)
iFLCr ≥ 100	30 (11.4%)			
Sens		25.7 (14.2, 42.1)	23.1 (14.5, 34.7)^b1^	18.7 (12.4, 27.1)
Spec		90.8 (86.3, 93.9)	92.4 (87.8, 95.3)^b2^	93.6 (88.6, 96.5)
iFLCr ≥ 100 and iFLC ≥ 100 mg/L	30 (11.4%)			
Sens		25.7 (14.2, 42.1)	23.1 (14.5, 34.7)^c1^	18.7 (12.4, 27.1)
Spec		90.8 (86.3, 93.9)	92.4 (87.8, 95.3)^c2^	93.6 (88.6, 96.5)
**Sebia cut-points**				
*N* (%) of subjects who progressed		36 (12.7%)	66 (23.2%)	111 (39.1%)
iFLCr ≥ 100	26 (9.2%)			
Sens		19.4 (9.7, 35.0)	16.7 (9.6, 27.5)^b1^	13.5 (8.4, 21.1)
Spec		92.3 (88.3, 95.0)	93.1 (88.9, 95.8)^b2^	93.6 (88.9, 96.4)
iFLCr ≥ 100 and iFLC ≥ 100 mg/L	25 (8.8%)			
Sens		16.7 (7.9, 31.9)	15.2 (8.5, 25.7)^c1^	12.6 (7.7, 20.0)
Spec		92.3 (88.3, 95.0)	93.1 (88.9, 95.8)^c2^	93.6 (88.9, 96.4)
**iFLCr cut-points for high-risk SMM,** ***n*** = **238 patients**				
**Freelite cut-points**^**d**^				
*N* (%) of subjects who progressed		23 (10.5%)	45 (20.6%)	80 (36.5%)
iFLCr > 20	72 (32.9%)			
Sens		52.2 (33.0, 70.8)	53.3 (39.0, 67.0)^e1, f1^	43.8 (33.5, 54.7)
Spec		69.4 (62.6, 75.4)	72.4 (65.3, 78.5)^e2,f2^	73.4 (65.5, 80.0)
**Sebia cut-points**				
*N* (%) of subjects who progressed		24 (10.1%)	46 (19.3%)	84 (35.3%)
iFLCr > 20	67 (28.2%)			
Sens		50.0 (31.4, 68.6)	45.7 (32.2, 59.9)^f1^	39.3 (29.5, 50.0)
Spec		74.3 (68.1, 79.7)	76.0 (69.5, 81.5)^f2^	77.9 (70.7, 83.7)

^a^21 did not have FreeLite involved FLC ratio (*n* = 263).

^b[1,2]^McNemar’s *p* value = 0.35 and 0.62 for paired sensitivity (*n* = 65) and specificity (*n* = 198), respectively, between FreeLite iFLCr ≥ 100 and Sebia iFLCr ≥ 100.

^c[1,2]^McNemar’s *p* value = 0.23 and 0.62 for paired sensitivity (*n* = 65) and specificity (*n* = 198), respectively, between FreeLite iFLCr ≥ 100 and FreeLite iFLC ≥ 100 mg/L and Sebia iFLCr ≥ 100 and Sebia iFLC ≥ 100 mg/L.

^d^*N* = 219 because 19 did not have FreeLite involved FLC ratio.

^e[1,2]^McNemar’s *p* value = 0.56 and 1.00 for paired sensitivity (*n* = 45) and specificity (*n* = 174), respectively, between FreeLite iFLCr > 20 and Sebia iFLCr > 16.

^f[1,2]^McNemar’s *p* value = 0.29 and 0.16 for paired sensitivity (*n* = 45) and specificity (*n* = 174), respectively, between FreeLite iFLCr > 20 and Sebia iFLCr > 20.

A plausible explanation for the excellent agreement for the use of the iFLCr to be generalized to the Sebia FLC reagents despite the methodological differences between the tests could lie in the predominantly monomeric structure of FLCs present in the SMM cohort of patients. It has been shown that FLCs may dimerize and form polymeric patterns, which can be pathogenic and are more common in AL and MM than in MGUS or SMM [8–10].

The second FLC threshold studied in this manuscript is the 20-20-20 system for SMM progression to MM, defined in 2018 [11]. In this system, a patient with SMM receives 1 risk point for each of the following: (1) M-spike > 20 g/L; (2) iFLCr > 20; and (3) BMPC > 20% [11–13]. This score has supplanted other scores for SMM risk and is used for trial stratification and eligibility. Because the FLC cut-points for both studies were based on the FreeLite assay, the goal was to identify comparable cut-points using the Sebia FLC. Applying the 2014 IMWG revised criteria for SMM and MM within the 284 patients cohort, 46 patients satisfied the revised definition of MM at baseline: ten based on BMPC, 30 based on FreeLite FLC definition of iFLC concentration ≥ 100 mg/L and iFLCr ≥ 100, ten based on MRI lesions. There was an overlap of MDE, including three patients meeting both BMPC and FLC criteria and one patient meeting both BMPC and MRI criteria. This left 238 patients to test the 20-20-20 SMM system. Applying the iFLCr greater than 20, this high-risk feature was present in 32.9% (72/219) and 28.2% (67/238) patients as measured by FreeLite and Sebia assays, respectively. A Sebia iFLCr > 20 can identify high-risk SMM, whose probability of progression is at least 50% within 60.4 (95% CI = 27.1–133.0) months compared to non-estimatable (NE) (95% CI = 150.7 - NE) months for iFLCr ≤20 (*p* = 0.001), similarly to FreeLite. Specificity of iFLCr greater than 20 for 24-month progression was comparable for FreeLite assay at 72.4% (126/174; 95% CI, [65.3%, 78.5%]) and the Sebia assay at 76.0% (146/192; 95% CI, [69.5%, 81.5%]) (Table 2), *p* = 0.16.

Cox proportional hazards models, adjusting for death as a competing risk, were used to evaluate the 20–20–20 system. The Sebia assay appears to perform as well in the 20-20-20 system as does the FreeLite assay (data shown in supplemental materials, c-statistic 0.668 for Sebia, and 0.671 for FreeLite). On univariate analysis, only the Sebia iFLCr was significant, but in separate multivariable models, neither the Sebia nor the FreeLite iFLCr nor the M-spike were significant, presumably due to reduced sample size, as the original 2018 study had 205 progression events (as compared to 100 in the current study) for Sebia cohort and 188 progression events (as compared to 91 in the current study) for FreeLite cohort, data shown in supplemental materials. The lack of statistical significance for FLC in the multivariable model shown in this present study should not challenge the utility of the system, given the smaller sample size of the study compared to the original larger IMWG cohort.

Having multiple assays to measure FLC available on the market will ultimately improve the diagnosis and monitoring of PCDs, enabling smaller laboratories to offer the testing with ease. The disadvantage that may arise is the lack of harmonization between assays which can lead to confusion and poor commutability of results across institutions. Our study suggests that it is acceptable to apply the existing FLC criteria used to define MM requiring therapy and to predict SMM risk of progression when using the Sebia assay, despite the analytical differences between assays.

We were able to show that the diagnostic value of the iFLCr ≥ 100 for MM progression in the SMM cohort used here is retained with the same specificity for Sebia FLC as the Freelite reagents in a cohort of 284 patients who fit the 2003 IMWG criteria for SMM. The 20-20-20 system established with the FreeLite reagents [12] to identify the high-risk progression of SMM after the new 2014 classification can also be applied when using Sebia FLC in our cohort of 238 patients. This commutability of the iFLCr cut-points in the SMM cohort is welcome in the hematology field and provides significant information to clinicians first seeing these patients and categorizing them as having a pre-malignant or malignant PCD.

## Supplementary information

Supplemental materials

## Data Availability

The data files for this study are stored in private Mayo Clinic servers and can be made available upon reasonable request to the authors.
