# Supplementary material for: Comparison of two free light chain assays: performance of the involved free light chain ratio and implications for diagnosis of multiple myeloma
Source: Blood Cancer J. 2022 Sep 2;12(9):127. doi: 10.1038/s41408-022-00722-5 (PMC9440004; doi:10.1038/s41408-022-00722-5)
Supplement: Supplementary file 1 — Supplemental materials [file 41408_2022_722_MOESM1_ESM.pdf]

Comparison of two free light chain assays: performance of the involved free light chain ratio and implications for diagnosis of multiple myeloma

Authors

Maria Alice V. Willrich, Ph.D.[1], David L. Murray, M.D., Ph.D.[1] Vincent Rajkumar, M.D.[2], Sandra C. Bryant, M.S.[3], Dirk Larson[3], Vanessa Pazdernik, M.S. [3], Melissa R. Snyder, Ph.D.[1], Robert A. Kyle, M.D. [2], and Angela Dispenzieri, M.D. [1,2]

Affiliations

1. Department of Laboratory Medicine and Pathology, Mayo Clinic, Rochester, MN. 2. Division of Hematology, Mayo Clinic, Rochester, MN 3. Department of Quantitative Health Sciences, Division of Clinical Trials and Biostatistics, Mayo Clinic, Rochester, MN

Text summary of contents in this file

1. Supplemental materials and methods
2. Analytical method comparison between FreeLite and Sebia FLC assays
    a. Supplemental Figure 1 with Passing Bablok linear regressions
3. Discussion of findings
4. Supplemental tables 1-3
5. References

Supplemental Materials

1. Supplemental Materials and Methods

Study Cohort

This study was reviewed and approved by the Mayo Clinic Institutional Review Board. Briefly, patients were identified by searching a computerized database and reviewing the medical records of patients who met the IMWG 2003 definition of SMM (1): >10% BMPCs and/or serum M-protein ≥30 g/L, plus absence of CRAB attributable to a PCD (calcium >0.25 mmol/L above the reference interval or > 2.75 mmol/L (>11.5 mg/dL), serum creatinine > 173 µmol/L (>2 mg/dL), hemoglobin 2 g/dL below the reference interval or <10 g/dL, or lytic lesions or diffuse osteopenia). From the original cohort of 586 of newly diagnosed SMM patients seen at the Mayo Clinic from 1976 to 2011 (2, 3) with authorization to review their medical records for research purposes, we excluded (n=302) those patients: 1) with no remaining stored serum sample within 30 days of SMM diagnosis; or 2) who had received prior chemotherapy at the time of SMM diagnosis (4). Events documenting progression to a malignant PCD (MM or amyloidosis, AL) and death were captured in our myeloma database via chart review and annual follow-up letters to patients.

Serum FLC assays

Aliquots of frozen stored serum were retrieved, and Sebia FLC tests were performed by ELISA. The ELISAs were run on a DS2 (Dynex Technologies, Chantilly, VA) automated platform and the program provided by Sebia. Per protocol, the sample is diluted 1:1 000 and incubated in a microplate where anti-kappa (or anti-lambda) specific antibodies are fixed. After washing the excess non-fixed mixture, anti-kappa (or anti-lambda) antiserum conjugated to peroxidase is pipetted onto the plate. After addition of the peroxidase substrate, the reaction is stopped with acid and the optical density is read by absorbance spectrophotometry at 450nm. The DS2 instrument can perform one plate at a time and the analytical run of one 96-well plate for kappa (or lambda) FLC takes between 2.5-3h. FreeLite serum FLC results were from the medical record or research database from the 2008/2013 studies. Briefly, FreeLite reagents use polyclonal sheep-antihuman antibodies to FLC, and testing was performed on Siemens BN2 (Siemens, Marburg, Germany) nephelometric analyzers. Reported values for both assays include kappa and lambda concentration along with a kappa/lambda ratio. For this study, the calculation of the iFLCr was done according to the isotype identified on immunofixation electrophoresis or the FreeLite serum FLC assay.

Other laboratory tests

As part of the patients' routine care, other laboratory analyses were ordered by the providers and information recorded in the myeloma research database. Information was collected for serum protein electrophoresis (Helena Laboratories, Belmont, TX), serum immunofixation (Sebia Inc) and BMPC measured on biopsy, when available within 30 days of the SMM diagnosis. Hemoglobin was retrieved from the complete blood count (CBC), creatinine and serum calcium from the central clinical laboratory tests at the time of the blood collection. Estimated glomerular filtration rate (eGFR) was calculated with the CKD-EPI equation using the standardized creatinine value (5, 6).

Statistical analysis

All patients were included for the population demographics. Data is summarized as median and range for continuous data and number and percent for categorical variables. Assays were compared using Passing-Bablok linear regression analysis for FLC measures. Estimates of sensitivity and specificity of the assays were compared at established cut-points for the FLC assays.

Progression was defined as a new clinical diagnosis of MM or AL after the SMM diagnosis. Time to progression was calculated based on MM or AL diagnosis date. Patients who were considered SMM in 2003 but who would have met 2014 criteria of MM requiring therapy (7) were excluded (n=46) for the analysis of the 20-20-20 system for SMM stratification. Cox proportional hazards models, adjusting for death as a competing risk, were used to assess univariate survival - measured as time to any progression from SMM

diagnosis, as well as to evaluate the 20-20-20 system. The c-statistic was used to assess the model's ability to predict any progression of SMM.

2. Brief analytical comparison between FreeLite and Sebia FLC assays

Serum free light chain assays by The Binding Site (FreeLite) and Sebia were compared. FreeLite kappa was missing for 20 patients, FreeLite lambda was missing for 20 patients, and there was overlap of missing data in both for 19 patients. Passing-Bablok linear regression is shown for kappa, lambda, kappa/lambda ratio and the iFLCr (**Supplemental Figure 1**). Slopes ranged from 0.634 for iFLCr to 0.747 for the kappa/lambda ratio.

Overall, when patients were categorized based on whether they had kappa or lambda restricted disease, the median involved FLC concentration and iFLCr were similar between FreeLite and Sebia, but due to the different analytical measurable ranges between assays, several of the comparisons between tests were statistically significant. In general, using the FreeLite assay, the values ran higher. The Sebia assay had a narrower range for kappa FLC and kappa iFLCr compared to FreeLite. In contrast, lambda FLC range was wider with Sebia (**Table 1, main manuscript content**).

3. Discussion of findings

Having multiple assays to measure FLC available on the market will ultimately improve the diagnosis and monitoring of PCDs, enabling smaller laboratories to offer the testing with ease. The disadvantage that may arise, as it happened with a few other laboratory analytes such as hemoglobin A1C (8-10) and prothrombin time (11), is the lack of harmonization between assays, which can lead to confusion and poor commutability of results across institutions**. Supplemental Table 2** demonstrates the different FLC assays on the market and their differences. A common question that clinical laboratories receive when reevaluating their FLC assay and considering a move to a different manufacturer is if the clinical decision points for these new reagents would be equivalent to the FreeLite assay. Our study suggests that it is acceptable to apply the existing FLC criteria used to define MM requiring therapy and to predict SMM risk of progression when using the Sebia assay.

In Scandinavia, Siemens has the largest market share of instruments in clinical laboratories, and groups using those reagents reported that the finding of an iFLC concentration of 100 mg/L or more could be applied to their N-Latex FLC assays as a risk factor for MGUS progression to MM (12, 13). The Sebia FLC ELISA method faces similar challenges as one of the newest reagents to reach clinical laboratories. Here we show that the iFLCr of 100 or more can be used for Sebia FLC assays with a sensitivity of 16.7% and specificity of 93.1%, virtually identical to FreeLite reagents, using a subset of the patients' samples originally used to define the cut-points in the IMWG 2014 guideline.

In a previous study with the Sebia FLC assay, newly diagnosed MM patients (177 patients with 368 samples) were tested simultaneously with FreeLite and Sebia FLC reagents (14). Authors verified the modest correlation between the methods that we also observed in this study and found a Pearson correlation coefficient r=0.713 for the iFLCr in diagnostics samples. Using a mathematical linear regression they concluded that the FreeLite cut-point of 100 could be converted to a cut-point of 16 using Sebia FLC (14). Another study compared 47 patients (31 newly diagnosed MM cases and 16 relapsed/refractory MM), with 177 samples (15). This group also verified the modest correlation between the assays when quantitatively comparing the individual concentrations of kappa and lambda or the iFLCr (15). To find comparable cut-points between the tests they used Cohen's Kappa coefficients and concluded The Sebia FLC cut-point of 100 was roughly equivalent to 20 using Sebia reagents, and the 20-20-20 system cut-point should be replaced for iFLCr of 8 using Sebia reagents (15). These two studies used a heterogenous group of active MM patients, and not a cohort of SMM to examine the iFLCr ≥ 100 cut-point. The findings from previous studies are difficult to compare to our current findings, as the study design from those is different from ours and from the original studies that defined the iFLCr ≥ 100 (4, 7). The use of conversion factors, while seeming practical for rebaselining subjects after a test change, are not the most appropriate tool to use in the clinical laboratory when the measurements are highly skewed and do not follow a Gaussian distribution. FreeLite and Sebia assays have a correlation coefficient (r) not exceeding 0.9, which means the assays show wide variance and results are not linearly correlated, especially for high concentrations of FLC, so often the conversion factor utilized may not represent the actual experimental test result.

Considering the use of the 20-20-20 system for high-risk SMM, the study shows that performance of the Sebia ELISA is at least as good as FreeLite, **Supplemental Table 3**.

In conclusion, despite the analytical differences between assays, we were able to show that the diagnostic value of the iFLCr ≥100 for MM progression in the SMM cohort used here is retained with the same specificity for Sebia as the Freelite reagents in a cohort of 284 patients who fit the 2003 IMWG criteria for SMM. The 20-20-20 system established with the FreeLite reagents (16) to identify high-risk progression of SMM after the new 2014 classification, can also be applied when using Sebia FLC tests in our cohort of 238 patients. This commutability of the iFLCr cut-points in the SMM cohort is welcome in the hematology field and provides significant information to clinicians first seeing these patients and categorizing them as having a pre-malignant or malignant PCD.

4. Supplemental Tables

Supplemental table 1. Progression in the smoldering multiple myeloma (SMM) cohort, classified using the 2003 IMWG criteria.

	Total (N=284)
**Progression to 1st malignant PCD within 1 yr.**	
No progression	248 (87.3%)
Progression	36 (12.7%)
**Progression to 1st malignant PCD within 2 yr.**	
No progression	218 (76.8%)
Progression	66 (23.2%)
**Progression to 1st malignant PCD within 3 yr.**	
No progression	191 (67.3%)
Progression	93 (32.7%)
**Progression to 1st malignant PCD within 4 yr.**	
No progression	182 (64.1%)
Progression	102 (35.9%)
**Progression to 1st malignant PCD within 5 yr.**	
No progression	173 (60.9%)
Progression	111 (39.1%)
**Type of progression to 1st malignant PCD**	
No progression	142 (50.0%)
Multiple Myeloma	137 (48.2%)
Light Chain Amyloidosis	5 (1.8%)
**Progression time to 1st malignant PCD, months**	
N	142
Median (Range)	25.4 (0.7-227.8)
**Time to last follow-up, months**	
N	284
Median (Range)	85.4 (0.1-462.9)

Supplemental Table 2. Various FLC assays available around the world

Manufacturer (Reagent name)	Different platforms using the reagents	Characteristic of the assay	iFLCr for a MDE and High risk SMM	Comments
The Binding Site (FreeLite)	Nephelometry<br>- Siemens BNII<br>- Siemens BN Prospec<br>- Beckmann Immage<br>Turbidimetry<br>- Optilite<br>- SpaPlus<br>- Roche cobas	Sheep anti-human polyclonal antibodies	MDE: ≥100 (4)<br>HR SMM: >20(16)	Testing may be performed by nephelometry or turbidimetry on the various platforms, different dilutions employed for each instrument. MDE and HR SMM defined with Freelite for BNII
Siemens Healthineers (N Latex)	Siemens BNII<br>Siemens BN Prospec<br>Atellica neph630	Cocktail of monoclonal antibodies	MDE: ≥70 (17)<br>HR SMM: 8 or 4.5 change (13)	All instruments are nephelometers
Sebia Inc. (Sebia FLC)	Any ELISA reader	Rabbit polyclonal antibodies	MDE: ≥100*<br>HR SMM: >20*	Same reagent set is versatile to be used with many automated or manual platforms
Roche (Diazyme)	Roche cobas	Polyclonal<br>(kappa: rabbit and goat; lambda: rabbit)	MDE: not available<br>HR SMM: not available	Turbidimetric method<br>No-cut-points are available for comparison with other assays
Seralite	Lateral flow immunoassay with a specific instrument<br>ADxLR5 absorbance reader	Cocktail of monoclonal antibodies	MDE: not available<br>HR SMM: not available	Lateral flow assay with quick turn-around-time

MDE, myeloma defining event; HR SMM, high-risk smoldering multiple myeloma; iFLCr, involved free light chain ratio. (*) Current publication

Supplemental Table 3. Univariate & multivariable analysis for risk factors for progression in smoldering multiple myeloma using 238 SMM patients*. Multivariable analysis using either the Sebia iFLCr of 20 or the FreeLite iFLCr of 20.

		**Univariate model****		**Sebia Multivariable model**[a]**			**FreeLite Multivariable model**[c]**		
**Risk factor**	**Time to progression, median (95% CI)**	**Hazard Ratio (95% CI)**	**p/c-stat**	**Hazard Ratio (95% CI)**	**p**	**c-stat**	**Hazard Ratio (95% CI)**	**p**	**c-stat**
Age, years		0.77 (0.53-1.11)	0.151/ 0.487	NI		0.668	NI		0.671
≤ 62 (n=102 [44.5%])	133.0 (59.6-NE)								
> 62 (n=136 [57.1%])	NE (108.2-NE)								
Sex		1.35 (0.93, 1.98)	0.138/ 0.547	NI			NI		
Male (n=132 [55.5%])	133.0 (57.2-NE)								
Female (n=106 [44.5%])	NE (106.5-NE)								
BMPC, %[a]		2.67 (1.76, 4.05)	**< 0.0001/ 0.643**	2.391 (1.567, 3.647)	**< 0.0001**		2.497 (1.625, 3.835)	**<0.0001**	
≤ 20% (n=96 [46.8%])	NE (227.8-NE)								
> 20% (n=109 [53.2%])	54.2 (33.0-85.3)								
Serum M-protein, g/L		1.79 (1.21, 2.64)	**0.006/ 0.570**	1.376 (0.899, 2.106)	0.142		1.516 (0.979, 2.348)	0.062	
≤ 20 g/L (n=104 [43.7%])	NE (NE-NE)								
> 20 g/L (n=134 [56.3%])	106.5 (59.6-NE)								
Sebia iFLCr		1.87 (1.27, 2.76)	**0.001/ 0.574**	1.383 (0.905, 2.116)	*0.134*		NI		
≤ 20 (n=171 [71.8%])	NE (150.7-NE)								
> 20 (n=67 [28.2%])	60.4 (27.1-133.0)								
FreeLite iFLCr[b]		1.48 (0.98-2.25)	0.063/ 0.577	NI			1.063 (0.675, 1.674)	0.794	
≤ 20 (n=147 [67.1%])	227.8 (119.8-NE)								
> 20 (n=72 [32.9%])	60.4 (26.3-NE)								
IgA, including biclonal		1.17 (0.78-1.76)	0.36/ 0.510	NI			NI		
Non-IgA (n=177 [74.4%])	NE (108.2-NE)								
IgA (n=61 [25.6%])	121.5 (57.2-NE)								
IgG, including biclonal		0.74 (0.49-1.10)	0.104/ 0.519	NI			NI		
Non-IgG (n=58 [24.4%])	103.7 (54.2-NE)								
IgG (n=180 [75.6%])	NE (119.8-NE)								

(*) 46 patients classified as active MM using the 2014 IMWG criteria for myeloma requiring therapy were excluded for this analysis. ** Cox proportional hazards model with adjustment for competing risk of death. [a]N=205 because 33 did not have BMPC. [b]N=219 because 19 did not have FreeLite involved FLC ratio. [c]N=188 because 50 did not have FreeLite involved FLC ratio and/or BMPC. CI=Confidence Interval, NE=Non-existent, NI=Not included

Supplemental Figure 1. Passing Bablok linear regression for kappa (1A), lambda (1B), involved FLC ratio (1C) and kappa/lambda free light chain ratio (1D), with FreeLite and Sebia assays for 284 SMM patients. Red traces represent the reference intervals for each assay. Gray traces show cut-points of 20 and 100 for the iFLCr.

1A kappa free light chain concentration, mg/L

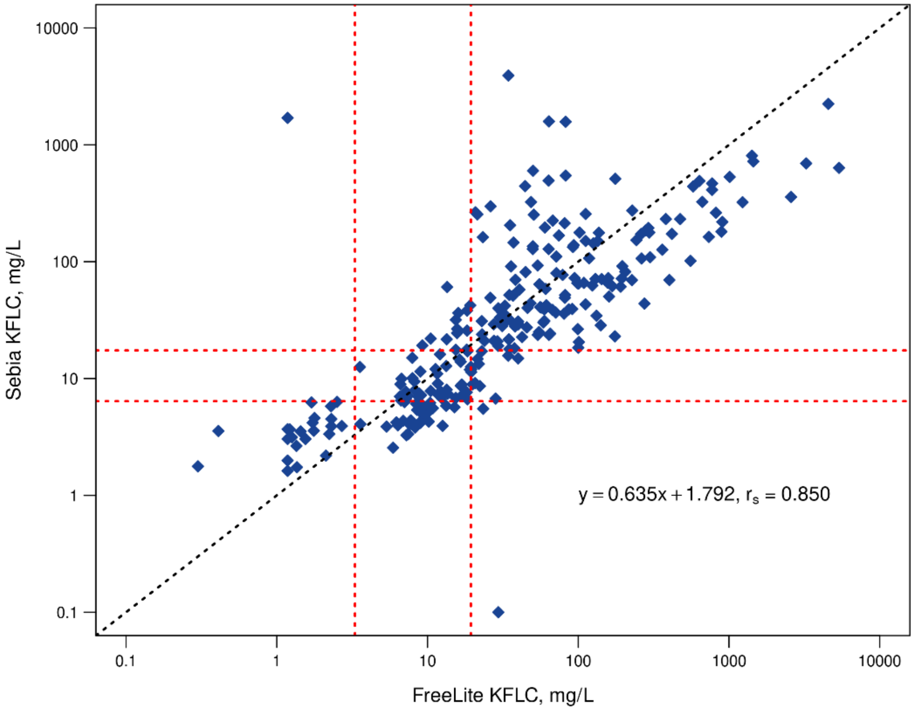

N=264 because missing FreeLite KFLC for 20 subjects.

1B lambda free light chain concentration, mg/L

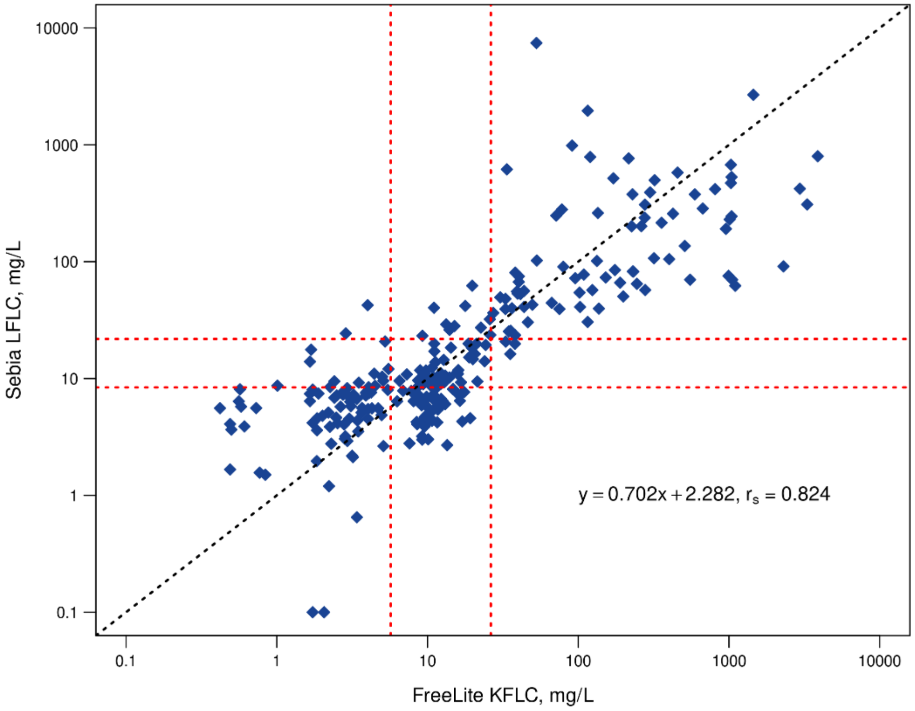

N=264 because missing FreeLite LFLC for 20 subjects.

1C involved to uninvolved free light chain ratio

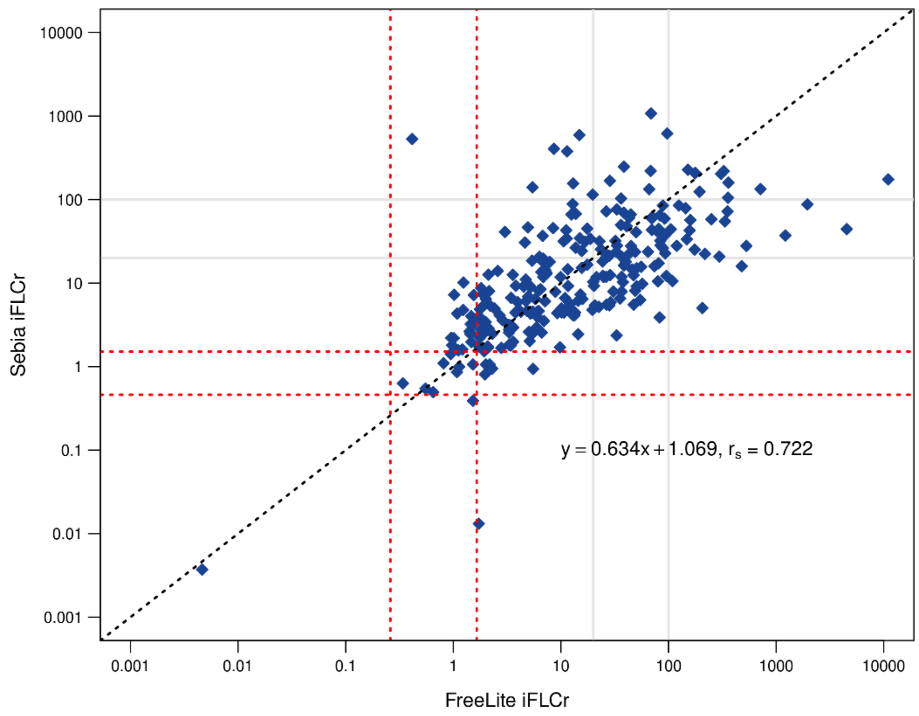

N=263 because missing FreeLite KFLC or LFLC for 21 subjects.

1D kappa/lambda free light chain ratio

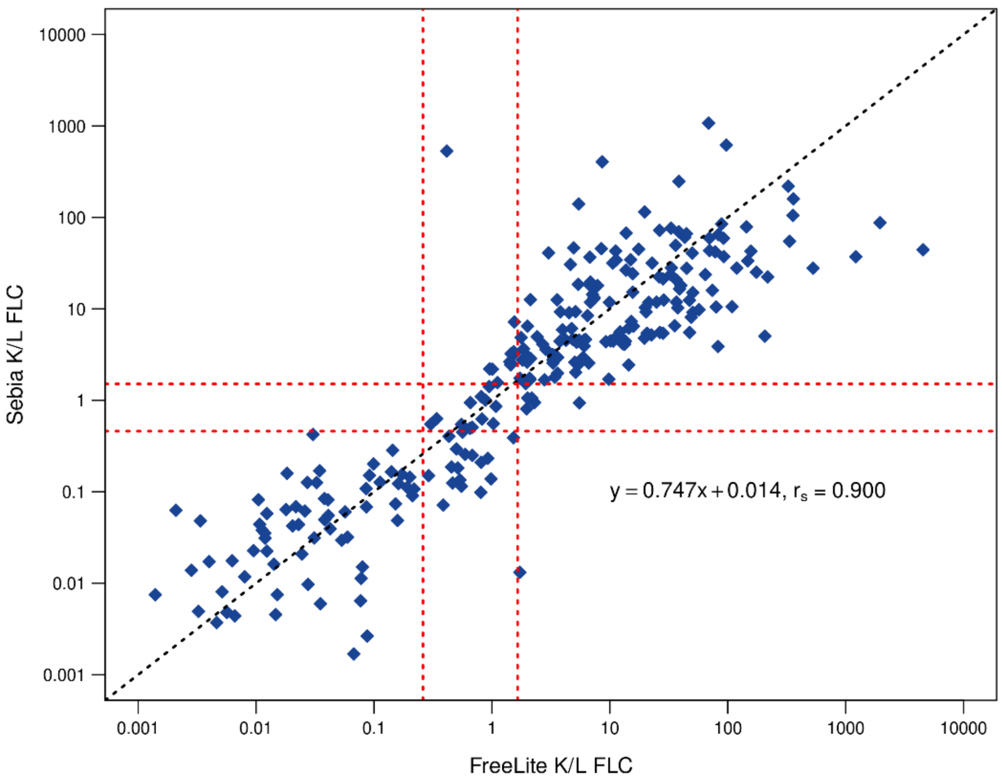

N=263 because missing FreeLite KFLC or LFLC for 21 subjects.

5. References

1. Criteria for the classification of monoclonal gammopathies, multiple myeloma and related disorders: a report of the International Myeloma Working Group. British journal of haematology. 2003;121(5):749-57.
2. Dispenzieri A, Kyle RA, Katzmann JA, Therneau TM, Larson D, Benson J, et al. Immunoglobulin free light chain ratio is an independent risk factor for progression of smoldering (asymptomatic) multiple myeloma. Blood. 2008;111(2):785-9.
3. Kyle RA, Remstein ED, Therneau TM, Dispenzieri A, Kurtin PJ, Hodnefield JM, et al. Clinical course and prognosis of smoldering (asymptomatic) multiple myeloma. N Engl J Med. 2007;356(25):2582-90.
4. Larsen JT, Kumar SK, Dispenzieri A, Kyle RA, Katzmann JA, Rajkumar SV. Serum free light chain ratio as a biomarker for high-risk smoldering multiple myeloma. Leukemia. 2013;27(4):941-6.
5. Levey AS, Stevens LA. Estimating GFR using the CKD Epidemiology Collaboration (CKD-EPI) creatinine equation: more accurate GFR estimates, lower CKD prevalence estimates, and better risk predictions. Am J Kidney Dis. 2010;55(4):622-7.
6. Valente MA, Hillege HL, Navis G, Voors AA, Dunselman PH, van Veldhuisen DJ, et al. The Chronic Kidney Disease Epidemiology Collaboration equation outperforms the Modification of Diet in Renal Disease equation for estimating glomerular filtration rate in chronic systolic heart failure. Eur J Heart Fail. 2014;16(1):86-94.
7. Rajkumar SV, Dimopoulos MA, Palumbo A, Blade J, Merlini G, Mateos MV, et al. International Myeloma Working Group updated criteria for the diagnosis of multiple myeloma. Lancet Oncol. 2014;15(12):e538-48.
8. Hanas R, John G, International Hb ACC. 2010 consensus statement on the worldwide standardization of the hemoglobin A1c measurement. Clin Chem. 2010;56(8):1362-4.
9. Little RR, Rohlfing C, Sacks DB. The National Glycohemoglobin Standardization Program: Over 20 Years of Improving Hemoglobin A1c Measurement. Clin Chem. 2019;65(7):839-48.
10. Little RR, Rohlfing CL, Sacks DB, National Glycohemoglobin Standardization Program Steering C. Status of hemoglobin A1c measurement and goals for improvement: from chaos to order for improving diabetes care. Clin Chem. 2011;57(2):205-14.
11. Dorgalaleh A, Favaloro EJ, Bahraini M, Rad F. Standardization of Prothrombin Time/International Normalized Ratio (PT/INR). Int J Lab Hematol. 2021;43(1):21-8.
12. Gran C, Liwing J, Wagner AK, Verhoek A, Gezin A, Alici E, et al. Comparative evaluation of involved free light chain and monoclonal spike as markers for progression from monoclonal gammopathy of undetermined significance to multiple myeloma. Am J Hematol. 2021;96(1):23-30.
13. Gran C, Luong V, Bruchfeld JB, Liwing J, Afram G, Lund J, et al. Dynamic follow-up of smoldering multiple myeloma identifies a subset of patients at high risk of progression. Am J Hematol. 2021;96(3):E63-E5.
14. Caillon H, Avet-Loiseau H, Attal M, Moreau P, Decaux O, Dejoie T. Comparison of Sebia Free Light Chain Assay With Freelite Assay for the Clinical Management of Diagnosis, Response, and Relapse Assessment in Multiple Myeloma. Clinical Lymphoma, Myeloma and Leukemia. 2019.

15. Schieferdecker A, Horber S, Ums M, Besemer B, Bokemeyer C, Peter A, et al. Comparison of three different serum-free light-chain assays-implications on diagnostic and therapeutic monitoring of multiple myeloma. Blood Cancer J. 2020;10(1):2.
16. Lakshman A, Rajkumar SV, Buadi FK, Binder M, Gertz MA, Lacy MQ, et al. Risk stratification of smoldering multiple myeloma incorporating revised IMWG diagnostic criteria. Blood Cancer J. 2018;8(6):59.
17. Henriot B, Rouger E, Rousseau C, Escoffre M, Sebillot M, Bendavid C, et al. Prognostic value of involved/uninvolved free light chain ratio determined by Freelite and N Latex FLC assays for identification of high-risk smoldering myeloma patients. Clin Chem Lab Med. 2019;57(9):1397-405.
